# New records of *Ticapimpla* Gauld, 1991 (Hymenoptera: Ichneumonidae: Pimplinae) from Brazil and French Guiana, with taxonomic notes

**DOI:** 10.3897/BDJ.7.e38141

**Published:** 2019-08-23

**Authors:** Diego G. Pádua, Ilari E. Sääksjärvi, Ricardo F. Monteiro, Marcio L. Oliveira

**Affiliations:** 1 Programa de Pós-Graduação em Entomologia, Instituto Nacional de Pesquisas da Amazônia - INPA, Manaus, Brazil Programa de Pós-Graduação em Entomologia, Instituto Nacional de Pesquisas da Amazônia - INPA Manaus Brazil; 2 Zoological Museum, Biodiversity Unit, University of Turku - UTU, Turku, Finland Zoological Museum, Biodiversity Unit, University of Turku - UTU Turku Finland; 3 Laboratório de Ecologia de Insetos, Depto. de Ecologia, Universidade Federal do Rio de Janeiro - UFRJ, Rio de Janeiro, Brazil Laboratório de Ecologia de Insetos, Depto. de Ecologia, Universidade Federal do Rio de Janeiro - UFRJ Rio de Janeiro Brazil

**Keywords:** Koinobiont, Amazonia, Neotropical, parasitoid wasps, *Polysphincta* genus-group, South America, tropical, rain forests, spiders.

## Abstract

**Background:**

We report the genus *Ticapimpla* Gauld, 1991 from French Guiana and the species *Ticapimpla amazonica* Palacio et al., *T. carinata* Palacio et al., *T. matamatae* Palacio et al. and *T. soinii* Palacio et al. from Brazilian Amazonia. The new discoveries suggest that the genus is widely distributed in Amazonian lowland rain forests. In addition, we diagnose and illustrate the males of *T. carinata* and *T. matamatae* for the first time. Short diagnoses and layer-photos for all the Amazonian species are provided.

**New information:**

The genus *Ticapimpla* is reported for the first time from French Guiana and the species *T. amazonica*, *T. carinata*, *T. matamatae* and *T. soinii* from Brazilian Amazonia. In addition, the males of *T. carinata* and *T. matamatae* are diagnosed and illustrated for the first time.

## Introduction

*Ticapimpla* Gauld, 1991 is a small Neotropical genus belonging to the *Polysphincta* group of genera. *Ticapimpla* species structurally resemble species of *Acrotaphus* Gravenhorst and *Hymenoepimecis* Viereck. These three genera share the following set of features: 1) occipital carina strongly raised and forming a flange-like protuberance (in most species), and 2) epomia absent (Gauld 1991, Gauld and Dubois 2006). *Ticapimpla* is easy to distinguish from *Acrotaphus* and *Hymenoepimecis* by having densely hirsute mesoscutum and the complete submetapleural carina (Palacio et al. 2010). In addition, the species of *Ticapimpla* are usually smaller than the species of *Acrotaphus* and *Hymenoepimecis*. Nothing is known about the biology of *Ticapimpla* but species of the *Polysphincta* group of genera are known to be koinobiont ectoparasitoids of active spiders (Gauld and Dubois 2006).

*Ticapimpla* includes five described species known from Costa Rica (Gauld 1991), Ecuador, Colombia and Peru (Palacio et al. 2010) and Brazil (Loffredo and Penteado-Dias 2008).

The aim of the paper is to report the genus for the first time from French Guiana and the species *T. amazonica* Palacio et al., 2010, *T. carinata* Palacio et al., 2010, *T. matamatae* Palacio et al., 2010 and *T. soinii* Palacio et al., 2010 from Brazilian Amazonia. In addition, we diagnose and illustrate the males of *T. carinata* and *T. matamatae* for the first time and provide diagnoses and layer-photos for all the Amazonian species.

## Materials and methods

The specimens examined in this paper are deposited in the following natural history collections: Invertebrate Collection of Instituto Nacional de Pesquisas da Amazônia, Manaus, Amazonas state, Brazil - INPA (Curator: Marcio L. Oliveira), Museu Paraense Emílio Goeldi, Belém, Pára state, Brazil - MPEG (Curator: Orlando T. Silveira), Museu de Zoologia da Universidade de São Paulo, São Paulo, São Paulo state, Brazil - MZUSP (Curator: Carlos R.F. Brandão) and Zoological Museum, Biodiversity Unit, University of Turku, Turku, Finland - ZMUT (Curator: Ilari E. Sääksjärvi).

Morphological terminology follow Gauld 1991, except for tarsal claws that follow Palacio et al. 2010.

Digital images were taken using a CANON DS126461 digital camera attached to an OLYMPUS SZX16 stereomicroscope. The captured images were assembled with the software Zerene Stacker (Version 1.04) and edited in Adobe Photoshop CS6.

The distributional data of the species was obtained from the labels and Palacio et al. (2010). The new country records of the genus were marked with “**” and new species records with “*”. The maps were made using SimpleMappr (https://www.simplemappr.net). In the distributional maps of *Ticapimpla* species, red circles indicate known records and yellow stars indicate new records. One symbol may represent many specimens.

## Taxon treatments

### Ticapimpla amazonica

Palacio, Broad, Sääksjärvi & Veijalainen, 2010

36BD851F5BE0569EB0B5C0BED17AD477

Ticapimpla amazonica Palacio, Broad, Sääksjärvi & Veijalainen, 2010 - Palacio et al. 2010.

#### Materials

- **Type status:** Other material. **Occurrence:** recordedBy: O. Morvan; individualCount: 1; sex: female; lifeStage: adult; **Location:** country: French Guiana; countryCode: GF; locality: M. de Kaw, Patawa; **Event:** eventDate: ii.2003; **Record Level:** institutionCode: ZMUT
- **Type status:** Other material. **Occurrence:** recordedBy: B. Klein; individualCount: 1; sex: female; lifeStage: adult; **Location:** country: Brazil; countryCode: BR; stateProvince: Amazonas; municipality: Manaus; locality: Reserva 1301, Fazenda Esteio, PDBFF; verbatimCoordinates: 02°23'03''S, 59°51'15''W; **Event:** samplingProtocol: Malaise trap; eventDate: 12.vi.1985; **Record Level:** institutionCode: INPA
- **Type status:** Other material. **Occurrence:** recordedBy: B. Klein; individualCount: 1; sex: female; lifeStage: adult; **Location:** country: Brazil; countryCode: BR; stateProvince: Amazonas; municipality: Manaus; locality: Reserva 1301, Fazenda Esteio, PDBFF; verbatimCoordinates: 02°23'03''S, 59°51'15''W; **Event:** samplingProtocol: Malaise trap; eventDate: 13.xi.1985; **Record Level:** institutionCode: INPA
- **Type status:** Other material. **Occurrence:** recordedBy: B. Klein; individualCount: 1; sex: female; lifeStage: adult; **Location:** country: Brazil; countryCode: BR; stateProvince: Amazonas; municipality: Manaus; locality: Reserva 1301, Fazenda Esteio, PDBFF; verbatimCoordinates: 02°23'03''S, 59°51'15''W; **Event:** samplingProtocol: Malaise trap; eventDate: 04.xii.1985; **Record Level:** institutionCode: INPA
- **Type status:** Other material. **Occurrence:** recordedBy: B. Klein; individualCount: 1; sex: female; lifeStage: adult; **Location:** country: Brazil; countryCode: BR; stateProvince: Amazonas; municipality: Manaus; locality: Reserva 1301, Fazenda Esteio, PDBFF; verbatimCoordinates: 02°23'03''S, 59°51'15''W; **Event:** samplingProtocol: Malaise trap; eventDate: 10.vii.1985; **Record Level:** institutionCode: INPA
- **Type status:** Other material. **Occurrence:** recordedBy: B. Klein; individualCount: 1; sex: male; lifeStage: adult; **Location:** country: Brazil; countryCode: BR; stateProvince: Amazonas; municipality: Manaus; locality: Reserva 1301, Fazenda Esteio, PDBFF; verbatimCoordinates: 02°23'03''S, 59°51'15''W; **Event:** samplingProtocol: Malaise trap; eventDate: 21.viii.1985; **Record Level:** institutionCode: INPA
- **Type status:** Other material. **Occurrence:** recordedBy: B. Klein; individualCount: 2; sex: 1 male and 1 female; lifeStage: adult; **Location:** country: Brazil; countryCode: BR; stateProvince: Amazonas; municipality: Manaus; locality: Reserva 1301, Fazenda Esteio, PDBFF; verbatimCoordinates: 02°23'03''S, 59°51'15''W; **Event:** samplingProtocol: Malaise trap; eventDate: 14.viii.1985; **Record Level:** institutionCode: INPA
- **Type status:** Other material. **Occurrence:** recordedBy: B. Klein; individualCount: 1; sex: male; lifeStage: adult; **Location:** country: Brazil; countryCode: BR; stateProvince: Amazonas; municipality: Manaus; locality: Reserva 1301, Fazenda Esteio, PDBFF; verbatimCoordinates: 02°23'03''S, 59°51'15''W; **Event:** samplingProtocol: Malaise trap; eventDate: 17.vii.1985; **Record Level:** institutionCode: INPA
- **Type status:** Other material. **Occurrence:** recordedBy: B. Klein; individualCount: 1; sex: male; lifeStage: adult; **Location:** country: Brazil; countryCode: BR; stateProvince: Amazonas; municipality: Manaus; locality: Reserva 1301, Fazenda Esteio, PDBFF; verbatimCoordinates: 02°23'03''S, 59°51'15''W; **Event:** samplingProtocol: Malaise trap; eventDate: 24.iv.1985; **Record Level:** institutionCode: INPA
- **Type status:** Other material. **Occurrence:** recordedBy: B. Klein; individualCount: 1; sex: female; lifeStage: adult; **Location:** country: Brazil; countryCode: BR; stateProvince: Amazonas; municipality: Manaus; locality: Reserva 1301, Fazenda Esteio, PDBFF; verbatimCoordinates: 02°23'03''S, 59°51'15''W; **Event:** samplingProtocol: Malaise trap; eventDate: 30.x.1985; **Record Level:** institutionCode: INPA
- **Type status:** Other material. **Occurrence:** recordedBy: B. Klein; individualCount: 1; sex: male; lifeStage: adult; **Location:** country: Brazil; countryCode: BR; stateProvince: Amazonas; municipality: Manaus; locality: Reserva 1113, Fazenda Esteio, PDBFF; verbatimCoordinates: 02°26'02''S, 59°51'15''W; **Event:** samplingProtocol: Malaise trap; eventDate: vii.1986; **Record Level:** institutionCode: INPA
- **Type status:** Other material. **Occurrence:** recordedBy: J.M.F. Ribeiro; individualCount: 1; sex: female; lifeStage: adult; **Location:** country: Brazil; countryCode: BR; stateProvince: Amazonas; municipality: Manaus; locality: Reserva Ducke, Igarapé Bolívia; **Event:** samplingProtocol: Malaise trap; eventDate: 10.ii.2003; **Record Level:** institutionCode: INPA

#### Diagnosis

This species can be distinguished from all other species of *Ticapimpla* by the combination of the following characters: 1) tarsal claw without auxiliary tooth, with a quadrangular flattened lobe, lobe with inner margin slightly convex (tarsal claw simple, without auxiliary tooth or preapical lobe in male); 2) epicnemial carina short, present only ventrally; 3) fore wing infumate (or very faintly infumate) with a weakly yellowish band between junction of vein *R*1 up to pterostigma until middle of the vein *M*; 4) hind leg orange, with distal 0.6 of tibia and tarsus black; 5) metasoma orange with tergites VI+ black; 6) occipital carina not forming a strongly raised flange in the occiput (Fig. 1a, Fig. 2a, Fig. 3a, Fig. 4a).

#### Distribution

Brazil*, Ecuador, French Guiana** and Peru (Fig. 5a).

### Ticapimpla carinata

Palacio, Broad, Sääksjärvi & Veijalainen, 2010

60D4160838795438BD74E3B9E7AFB186

Ticapimpla carinata Palacio, Broad, Sääksjärvi & Veijalainen, 2010 - Palacio et al. 2010

#### Materials

- **Type status:** Other material. **Occurrence:** recordedBy: J. Cerda; individualCount: 1; sex: male; lifeStage: adult; **Location:** country: French Guiana; countryCode: GF; locality: M. de Kaw; **Event:** eventDate: ix.2002; **Record Level:** institutionCode: ZMUT
- **Type status:** Other material. **Occurrence:** recordedBy: B. Klein; individualCount: 1; sex: female; lifeStage: adult; **Location:** country: Brazil; countryCode: BR; stateProvince: Amazonas; municipality: Manaus; locality: Reserva 1301, Fazenda Esteio, PDBFF; verbatimCoordinates: 02°23'03''S, 59°51'15''W; **Event:** samplingProtocol: Malaise trap; eventDate: 15.v.1985; **Record Level:** institutionCode: INPA
- **Type status:** Other material. **Occurrence:** recordedBy: B. Klein; individualCount: 1; sex: female; lifeStage: adult; **Location:** country: Brazil; countryCode: BR; stateProvince: Amazonas; municipality: Manaus; locality: Reserva 1301, Fazenda Esteio, PDBFF; verbatimCoordinates: 02°23'03''S, 59°51'15''W; **Event:** samplingProtocol: Malaise trap; eventDate: 13.xi.1985; **Record Level:** institutionCode: INPA
- **Type status:** Other material. **Occurrence:** recordedBy: B. Klein; individualCount: 1; sex: male; lifeStage: adult; **Location:** country: Brazil; countryCode: BR; stateProvince: Amazonas; municipality: Manaus; locality: Reserva 1301, Fazenda Esteio, PDBFF; verbatimCoordinates: 02°23'03''S, 59°51'15''W; **Event:** samplingProtocol: Malaise trap; eventDate: 04.xii.1985; **Record Level:** institutionCode: INPA
- **Type status:** Other material. **Occurrence:** recordedBy: B. Klein; individualCount: 1; sex: female; lifeStage: adult; **Location:** country: Brazil; countryCode: BR; stateProvince: Amazonas; municipality: Manaus; locality: Reserva 1301, Fazenda Esteio, PDBFF; verbatimCoordinates: 02°23'03''S, 59°51'15''W; **Event:** samplingProtocol: Malaise trap; eventDate: 30.x.1985; **Record Level:** institutionCode: INPA
- **Type status:** Other material. **Occurrence:** recordedBy: B. Klein; individualCount: 1; sex: female; lifeStage: adult; **Location:** country: Brazil; countryCode: BR; stateProvince: Amazonas; municipality: Manaus; locality: Reserva 1301, Fazenda Esteio, PDBFF; verbatimCoordinates: 02°23'03''S, 59°51'15''W; **Event:** samplingProtocol: Malaise trap; eventDate: 21.viii.1985; **Record Level:** institutionCode: INPA
- **Type status:** Other material. **Occurrence:** recordedBy: B. Klein; individualCount: 1; sex: male; lifeStage: adult; **Location:** country: Brazil; countryCode: BR; stateProvince: Amazonas; municipality: Manaus; locality: Reserva 1301, Fazenda Esteio, PDBFF; verbatimCoordinates: 02°23'03''S, 59°51'15''W; **Event:** samplingProtocol: Malaise trap; eventDate: 19.vi.1985; **Record Level:** institutionCode: INPA
- **Type status:** Other material. **Occurrence:** recordedBy: B. Klein; individualCount: 2; sex: female; lifeStage: adult; **Location:** country: Brazil; countryCode: BR; stateProvince: Amazonas; municipality: Manaus; locality: Reserva 1113, Fazenda Esteio, PDBFF; verbatimCoordinates: 02°26'02''S, 59°51'15''W; **Event:** samplingProtocol: Malaise trap; eventDate: iv.1986; **Record Level:** institutionCode: INPA
- **Type status:** Other material. **Occurrence:** recordedBy: B. Klein; individualCount: 1; sex: male; lifeStage: adult; **Location:** country: Brazil; countryCode: BR; stateProvince: Amazonas; municipality: Manaus; locality: Reserva 1208, Fazenda Esteio, PDBFF; verbatimCoordinates: 02°22'34''S, 59°52'39''W; **Event:** samplingProtocol: Malaise trap; eventDate: 16.vii.1985; **Record Level:** institutionCode: INPA
- **Type status:** Other material. **Occurrence:** recordedBy: B. Klein; individualCount: 1; sex: male; lifeStage: adult; **Location:** country: Brazil; countryCode: BR; stateProvince: Amazonas; municipality: Manaus; locality: Reserva 1208, Fazenda Esteio, PDBFF; verbatimCoordinates: 02°22'34''S, 59°52'39''W; **Event:** samplingProtocol: Malaise trap; eventDate: 11.vi.1985; **Record Level:** institutionCode: INPA
- **Type status:** Other material. **Occurrence:** recordedBy: B. Klein; individualCount: 1; sex: male; lifeStage: adult; **Location:** country: Brazil; countryCode: BR; stateProvince: Amazonas; municipality: Manaus; locality: Reserva 1208, Fazenda Esteio, PDBFF; verbatimCoordinates: 02°22'34''S, 59°52'39''W; **Event:** samplingProtocol: Malaise trap; eventDate: 06.viii.1985; **Record Level:** institutionCode: INPA
- **Type status:** Other material. **Occurrence:** recordedBy: B. Klein; individualCount: 1; sex: male; lifeStage: adult; **Location:** country: Brazil; countryCode: BR; stateProvince: Amazonas; municipality: Manaus; locality: Reserva 1208, Fazenda Esteio, PDBFF; verbatimCoordinates: 02°22'34''S, 59°52'39''W; **Event:** samplingProtocol: Malaise trap; eventDate: 03.xii.1985; **Record Level:** institutionCode: INPA
- **Type status:** Other material. **Occurrence:** recordedBy: L. Ulisses; individualCount: 1; sex: male; lifeStage: adult; **Location:** country: Brazil; countryCode: BR; stateProvince: Amazonas; municipality: Manaus; locality: Reserva Ducke; **Event:** samplingProtocol: Malaise trap; eventDate: 23.ix.1986; **Record Level:** institutionCode: INPA
- **Type status:** Other material. **Occurrence:** recordedBy: J.M.F. Ribeiro; J. Vidal & J.A. Vidal; individualCount: 1; sex: female; lifeStage: adult; **Location:** country: Brazil; countryCode: BR; stateProvince: Amazonas; municipality: Manaus; locality: Reserva Ducke, Igarapé Uberé; **Event:** samplingProtocol: Malaise trap; eventDate: vii.2001; **Record Level:** institutionCode: INPA
- **Type status:** Other material. **Occurrence:** recordedBy: R. Ale-Rocha; J.F. Vidal & A.P. Marques; individualCount: 1; sex: female; lifeStage: adult; **Location:** country: Brazil; countryCode: BR; stateProvince: Amazonas; municipality: Manaus; locality: Reserva Ducke, Mata; **Event:** samplingProtocol: Fruit bait; eventDate: 23-27.xiii.2000 [sic]; **Record Level:** institutionCode: INPA
- **Type status:** Other material. **Occurrence:** recordedBy: R. Ale-Rocha and team; individualCount: 1; sex: female; lifeStage: adult; **Location:** country: Brazil; countryCode: BR; stateProvince: Amazonas; locality: Rio Nhamundá, Cuipiranga; verbatimCoordinates: 01°53'58''S, 57°02'59''W; **Event:** samplingProtocol: Suspense trap; eventDate: 20-23.v.2008; **Record Level:** institutionCode: INPA

#### Description

**Male** (Fig. 4b). Similar to female in structure and coloration, but body about 5.0–7.0 mm; tarsal claw simple, without auxiliary tooth or preapical lobe.

#### Diagnosis

This species can be distinguished from all other species of *Ticapimpla* by the combination of the following characters: 1) tarsal claw with auxiliary tooth and with inner margin strongly concave (tarsal claw simple, without auxiliary tooth or preapical lobe in male); 2) epicnemial carina short, present only ventrally; 3) fore wing infumate, with a weakly yellowish band between junction of vein *R*1 up to pterostigma until middle of the vein *M* or very faintly yellowish with apex and area adjacent to pterostigma of fore wing clearly infumate; 4) hind leg orange, with distal 0.6 of tibia and tarsus black; 5) metasoma orange with tergites VI+ black; 6) occipital carina forming a strongly raised flange in the occiput (Fig. 1b, Fig. 2b, Fig. 3b, Fig. 4b)

#### Distribution

Brazil*, Colombia, French Guiana** and Peru (Fig. 5b).

### Ticapimpla matamatae

Palacio, Broad, Sääksjärvi & Veijalainen, 2010

7250F7AA6EB6595DAC79FAD0E24B04CE

Ticapimpla matamatae Palacio, Broad, Sääksjärvi & Veijalainen, 2010 - Palacio et al. 2010

#### Materials

- **Type status:** Other material. **Occurrence:** recordedBy: B. Klein; individualCount: 1; sex: female; lifeStage: adult; **Location:** country: Brazil; countryCode: BR; stateProvince: Amazonas; municipality: Manaus; locality: Reserva 1301, Fazenda Esteio, PDBFF; verbatimCoordinates: 02°23'03''S, 59°51'15''W; **Event:** samplingProtocol: Malaise trap; eventDate: 17.vii.1985; **Record Level:** institutionCode: INPA
- **Type status:** Other material. **Occurrence:** recordedBy: K. Schoeninger; individualCount: 1; sex: female; lifeStage: adult; **Location:** country: Brazil; countryCode: BR; stateProvince: Amazonas; municipality: Manaus; locality: EMBRAPA, Cultivo de Guaraná orgânico, Ponto Mata; verbatimCoordinates: 02°53'29.14''S, 59°58'45.80''W; **Event:** samplingProtocol: Malaise trap; eventDate: 11.x.2012; **Record Level:** institutionCode: INPA
- **Type status:** Other material. **Occurrence:** recordedBy: J.M.F. Ribeiro; individualCount: 1; sex: female; lifeStage: adult; **Location:** country: Brazil; countryCode: BR; stateProvince: Amazonas; municipality: Manaus; locality: Reserva Ducke, Igarapé Bolívia; **Event:** samplingProtocol: Malaise trap; eventDate: 10.ii.2003; **Record Level:** institutionCode: INPA
- **Type status:** Other material. **Occurrence:** recordedBy: J.A. Rafael et al.; individualCount: 1; sex: male; lifeStage: adult; **Location:** country: Brazil; countryCode: BR; stateProvince: Amazonas; locality: Rio Nhamundá, Cuipiranga; verbatimElevation: 22 m; **Event:** samplingProtocol: Malaise trap; eventDate: 20-23.v.2008; **Record Level:** institutionCode: INPA
- **Type status:** Other material. **Occurrence:** recordedBy: O. Silveira & J. Dias; individualCount: 1; sex: female; lifeStage: adult; **Location:** country: Brazil; countryCode: BR; stateProvince: Pará; municipality: Melgaço; locality: ECFPn, Percurso 1, Trilha 3, Tijucaquara; **Event:** eventDate: 24.vi.1998; **Record Level:** institutionCode: MPEG
- **Type status:** Other material. **Occurrence:** recordedBy: without collector; individualCount: 1; sex: male; lifeStage: adult; **Location:** country: Brazil; countryCode: BR; stateProvince: Pará; municipality: Altamira; locality: Rio Xingú, A1-Itapuama, S. Antônio; **Event:** samplingProtocol: Malaise trap; eventDate: 19-23.vii.2008; **Record Level:** institutionCode: MPEG
- **Type status:** Other material. **Occurrence:** recordedBy: B. Malkin; individualCount: 1; sex: female; lifeStage: adult; **Location:** country: Brazil; countryCode: BR; stateProvince: Maranhăo; locality: Aldeia Maracaçumé, Rio Maracaçumé; **Event:** eventDate: v.1963; **Record Level:** institutionCode: MZUSP

#### Description

**Male** (Fig. 4c). Similar to female in structure and coloration, but body about 7.0 mm; tarsal claw simple, without auxiliary tooth or preapical lobe.

#### Diagnosis

This species can be distinguished from all other species of *Ticapimpla* by the combination of the following characters: 1) tarsal claw with a preapical auxiliary tooth (tarsal claw simple, without auxiliary tooth or preapical lobe in male); 2) epicnemial carina entirely absent; 3) fore wing yellowish, with apex infumate and with an infumate median band extending backwards from anterior margin, just proximal to the pterostigma, right until the junction of the veins *Rs&M* with *cu-a*; 4) hind leg orange, with femur, tibia and tarsus black; 5) metasoma orange with tergites V+ or VI+ black; 6) occipital carina forming a strongly raised flange in the occiput (Fig. 1c, Fig. 2c, Fig. 3c, Fig. 4c).

#### Distribution

Brazil* and Colombia (Fig. 5c).

### Ticapimpla soinii

Palacio, Broad, Sääksjärvi & Veijalainen, 2010

D22FD6E9A8945C979B22D5C3AF0BF186

Ticapimpla soinii Palacio, Broad, Sääksjärvi & Veijalainen, 2010 - Palacio et al. 2010

#### Materials

- **Type status:** Other material. **Occurrence:** recordedBy: K. Schoeninger; individualCount: 1; sex: male; lifeStage: adult; **Location:** country: Brazil; countryCode: BR; stateProvince: Amazonas; municipality: Manaus; locality: EMBRAPA, Cultivo de Guaraná convencional, Point Mata; **Event:** samplingProtocol: Moerick trap; eventDate: 26.x.2012; **Record Level:** institutionCode: INPA
- **Type status:** Other material. **Occurrence:** recordedBy: J.M.F. Ribeiro & J. Vidal; individualCount: 1; sex: female; lifeStage: adult; **Location:** country: Brazil; countryCode: BR; stateProvince: Amazonas; municipality: Manaus; locality: Reserva Ducke, Igarapé Uberé; **Event:** samplingProtocol: Malaise trap; eventDate: vi.2003; **Record Level:** institutionCode: INPA
- **Type status:** Other material. **Occurrence:** recordedBy: J.A. Vidal & J. Vidal; individualCount: 1; sex: female; lifeStage: adult; **Location:** country: Brazil; countryCode: BR; stateProvince: Amazonas; municipality: Manaus; locality: Reserva Ducke, Igarapé Ipiranga; **Event:** samplingProtocol: Malaise trap; eventDate: 31.xii.2002; **Record Level:** institutionCode: INPA

#### Diagnosis

This species can be distinguished from all other species of *Ticapimpla* by the combination of the following characters: 1) tarsal claw without auxiliary tooth, instead with a preapical, flattened lobe, lobe with inner margin concave (tarsal claw simple, without auxiliary tooth or preapical lobe in male); 2) epicnemial carina short, present only ventrally; 3) fore wing very faintly yellowish, the fore wing with apex and area adjacent to pterostigma infumate; 4) hind leg orange, with distal 0.6 of tibia and tarsus black; 5) metasoma orange with tergites VI+ black; 6) occipital carina forming a strongly raised flange in the occiput (Fig. 1d, Fig. 2d, Fig. 3d, Fig. 4d).

#### Distribution

Brazil*, Colombia, Ecuador and Peru (Fig. 5d).

## Discussion

Palacio et al. (2010) proposed that *Ticapimpla* is a taxon of South American origin. Only one species, *T. vilmae* Gauld, 1991, is known from Central America. In South America, the genus is best represented in Amazonian lowland rain forests.

Palacio et al. (2010) reported most of the specimens from Western Amazonia. The present study shows that the genus is widely distributed in Amazonia. Interestingly, despite of studying a vast number of new ichneumonid samples collected from Amazonia, we have not been able to discover new species of *Ticapimpla*. This indicates that this genus is among the best known Pimplinae genera in the Neotropical region.

## Supplementary Material

XML Treatment for Ticapimpla amazonica

XML Treatment for Ticapimpla carinata

XML Treatment for Ticapimpla matamatae

XML Treatment for Ticapimpla soinii

## Figures and Tables

**Figure 1a.**
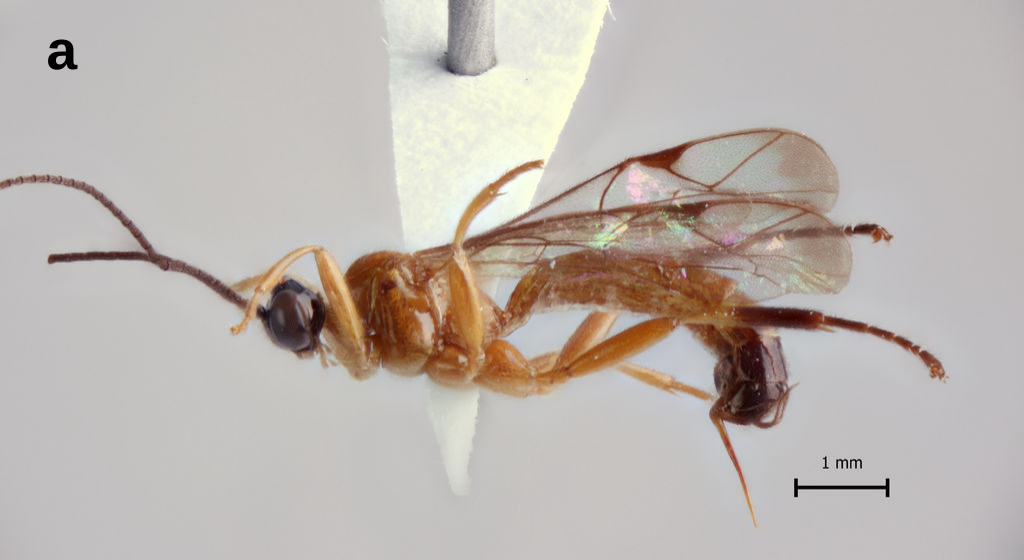
*T. amazonica* Palacio et al., 2010

**Figure 1b.**
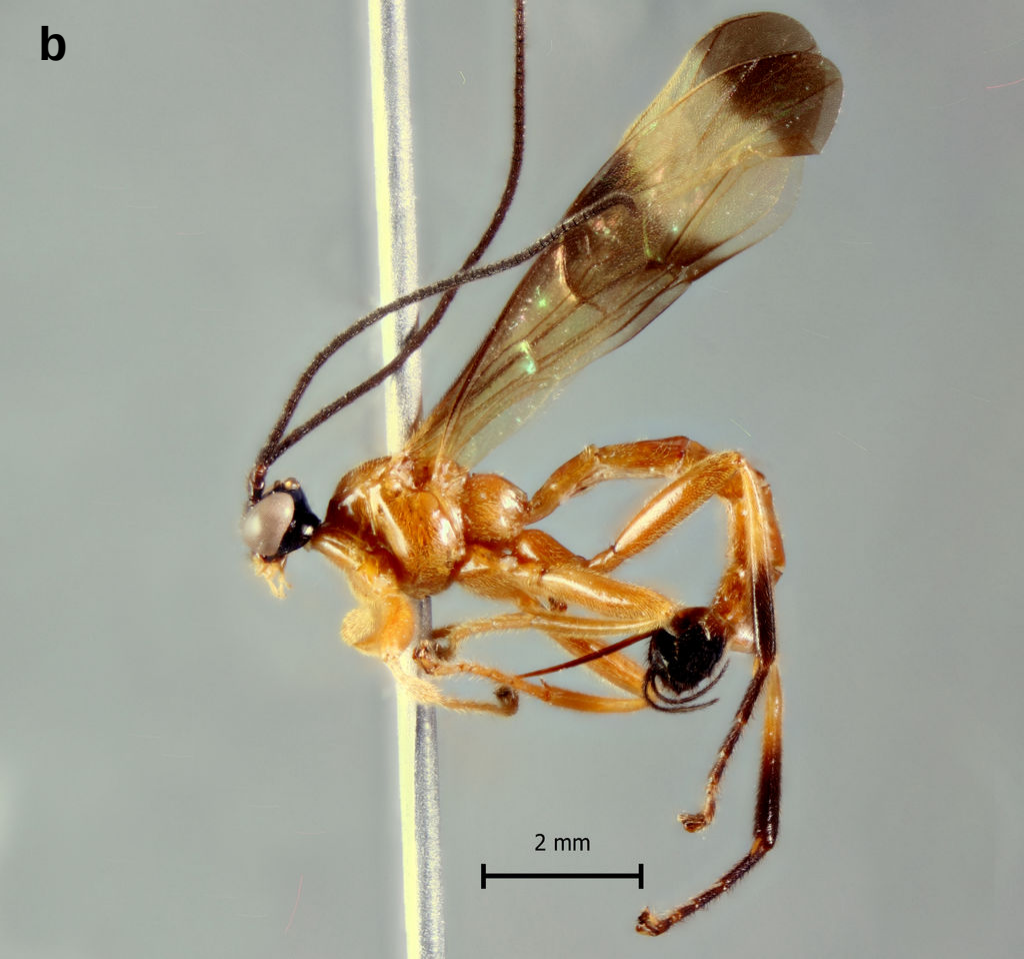
*T. carinata* Palacio et al., 2010

**Figure 1c.**
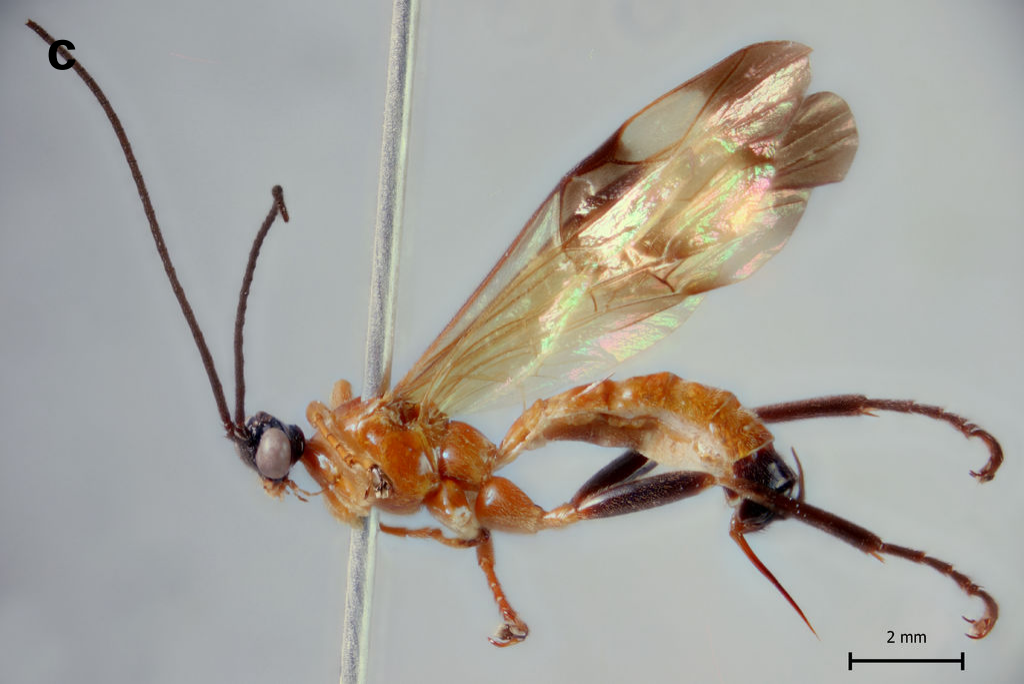
*T. matamatae* Palacio et al., 2010

**Figure 1d.**
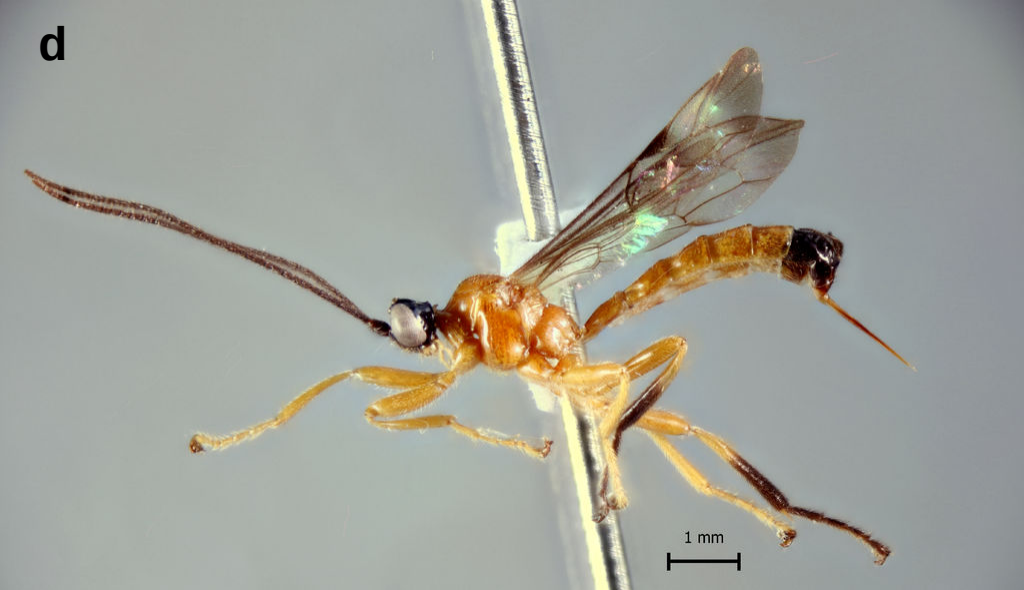
*T. soinii* Palacio et al., 2010.

**Figure 2a.**
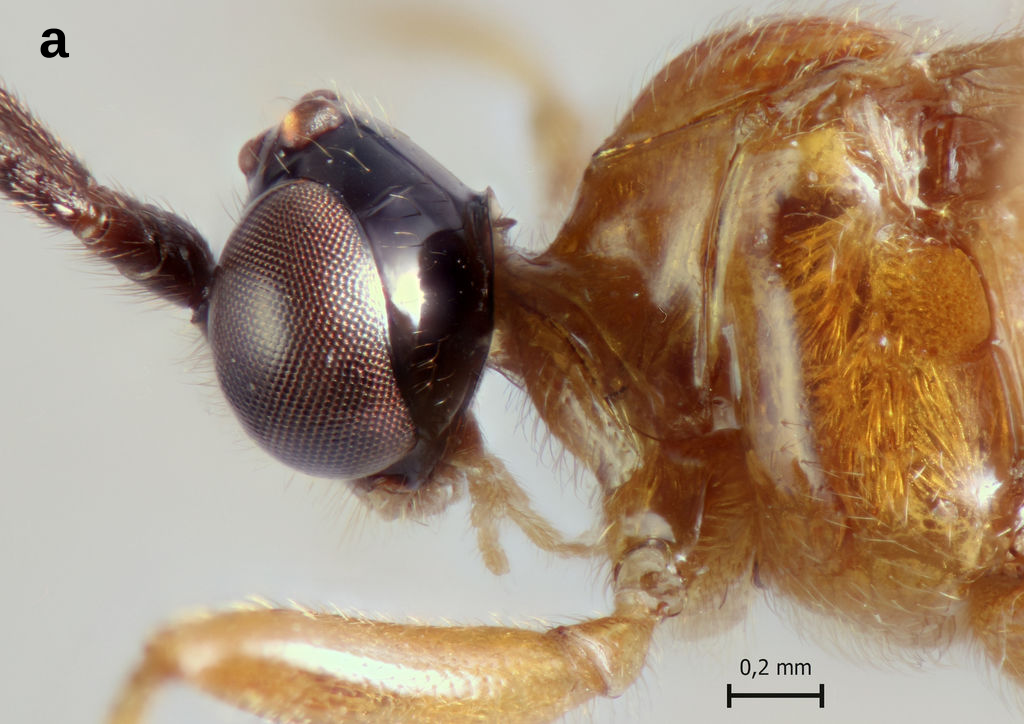
*T. amazonica* Palacio et al., 2010

**Figure 2b.**
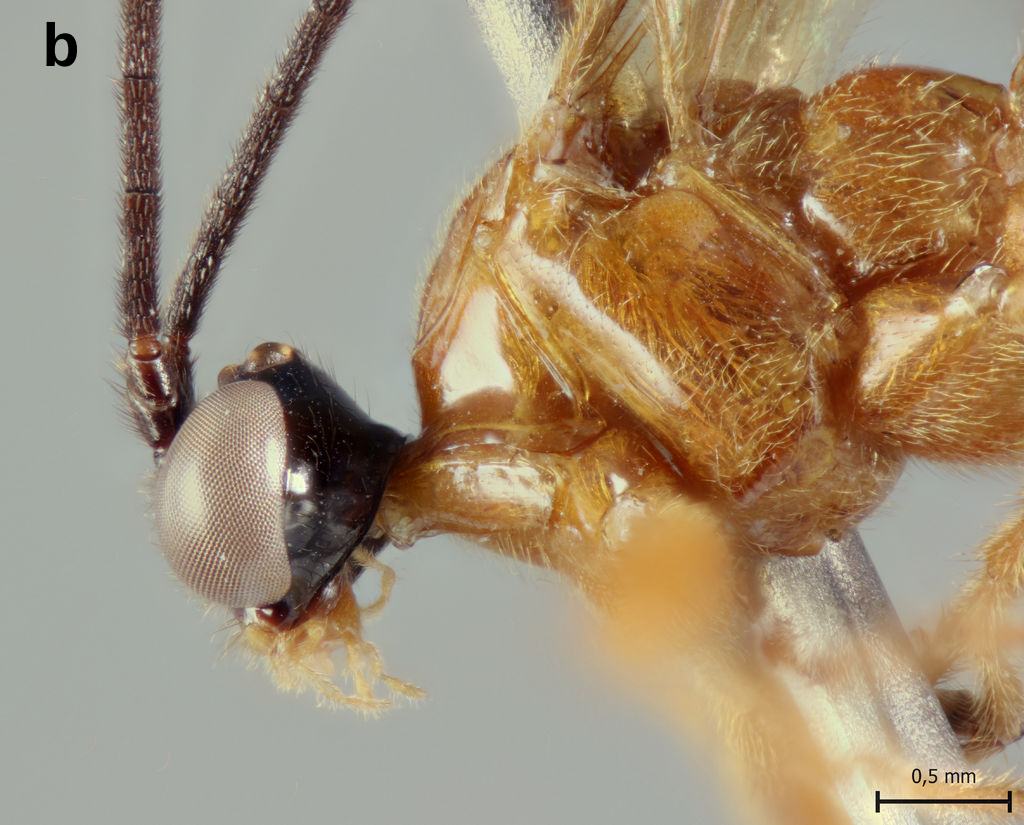
*T. carinata* Palacio et al., 2010;

**Figure 2c.**
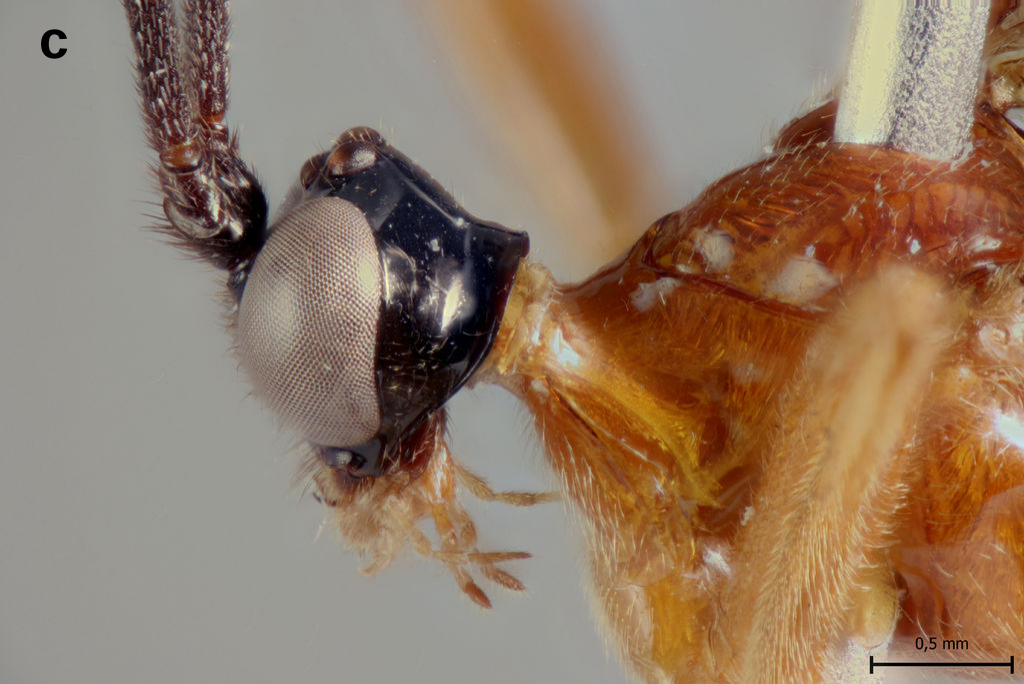
*T. matamatae* Palacio et al., 2010

**Figure 2d.**
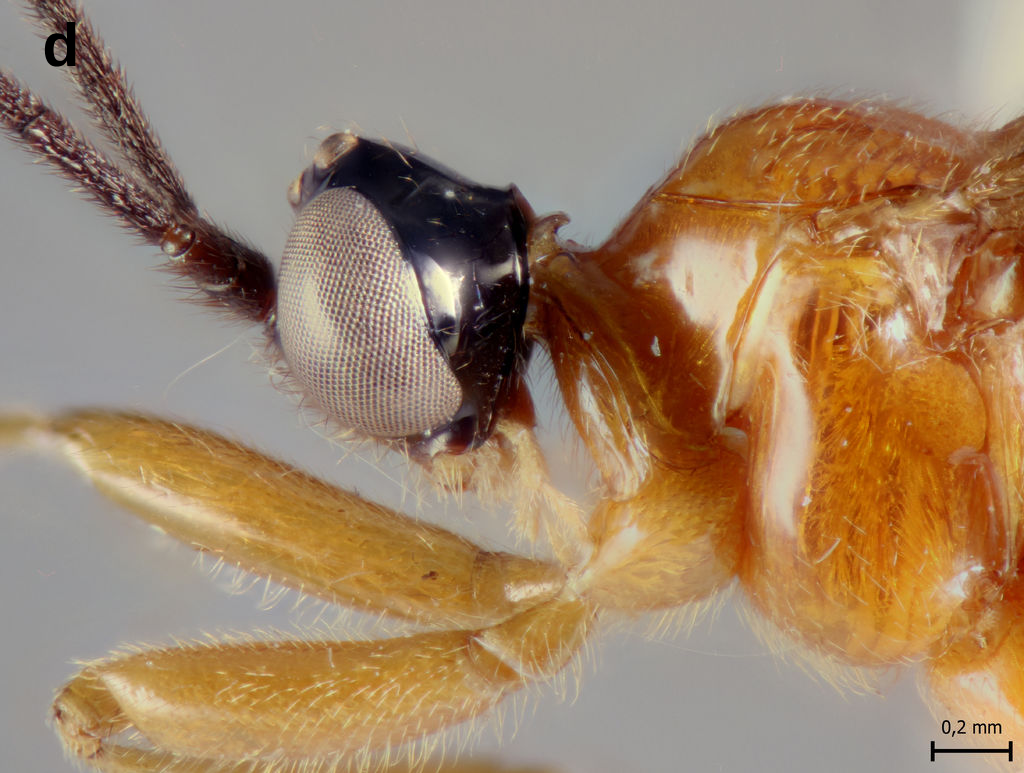
*T. soinii* Palacio et al., 2010.

**Figure 3a.**
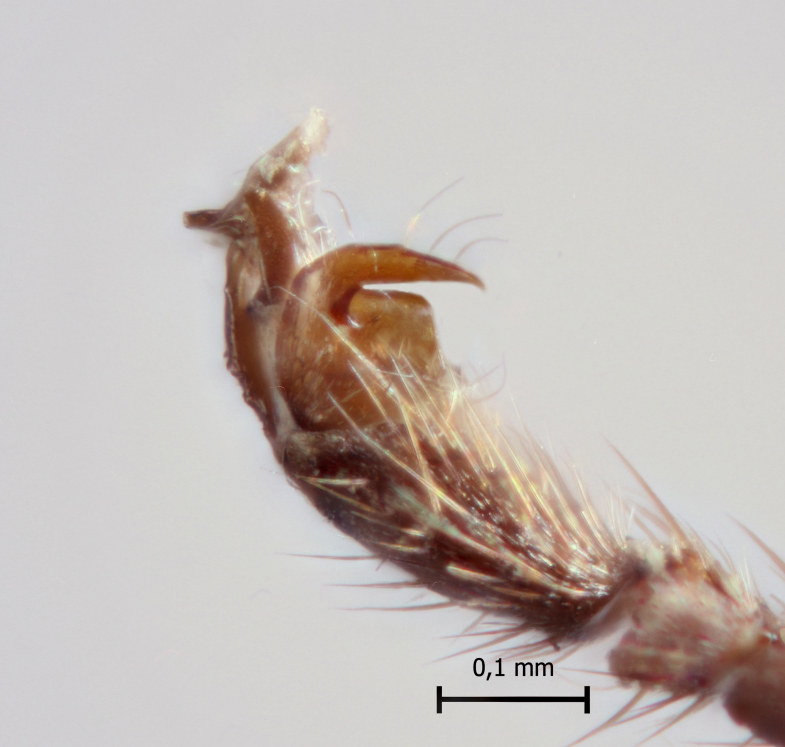
*T. amazonica* Palacio et al., 2010

**Figure 3b.**
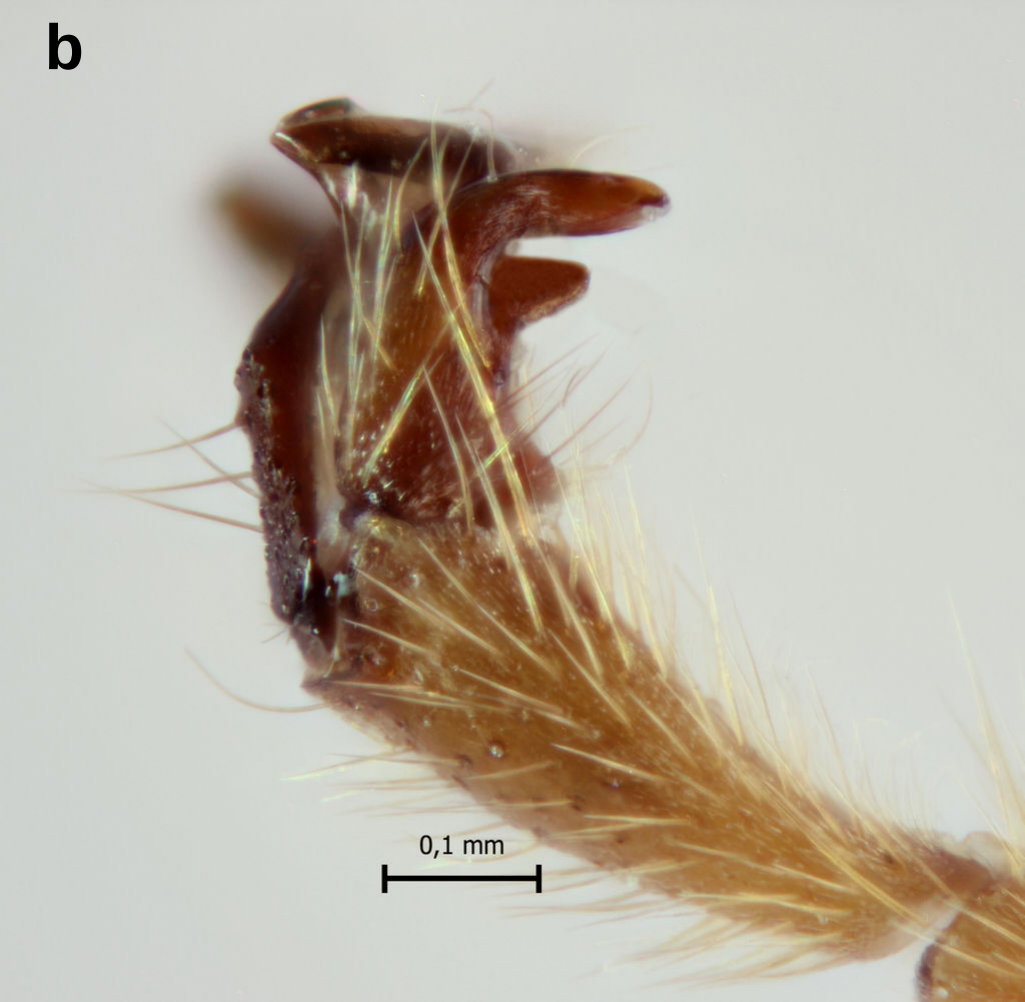
*T. carinata* Palacio et al., 2010

**Figure 3c.**
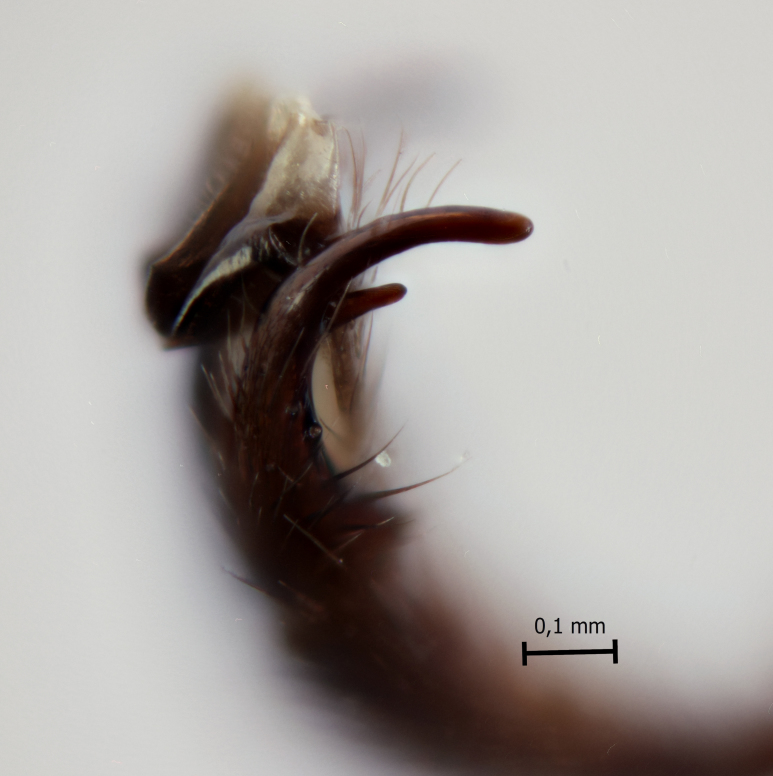
*T. matamatae* Palacio et al., 2010

**Figure 3d.**
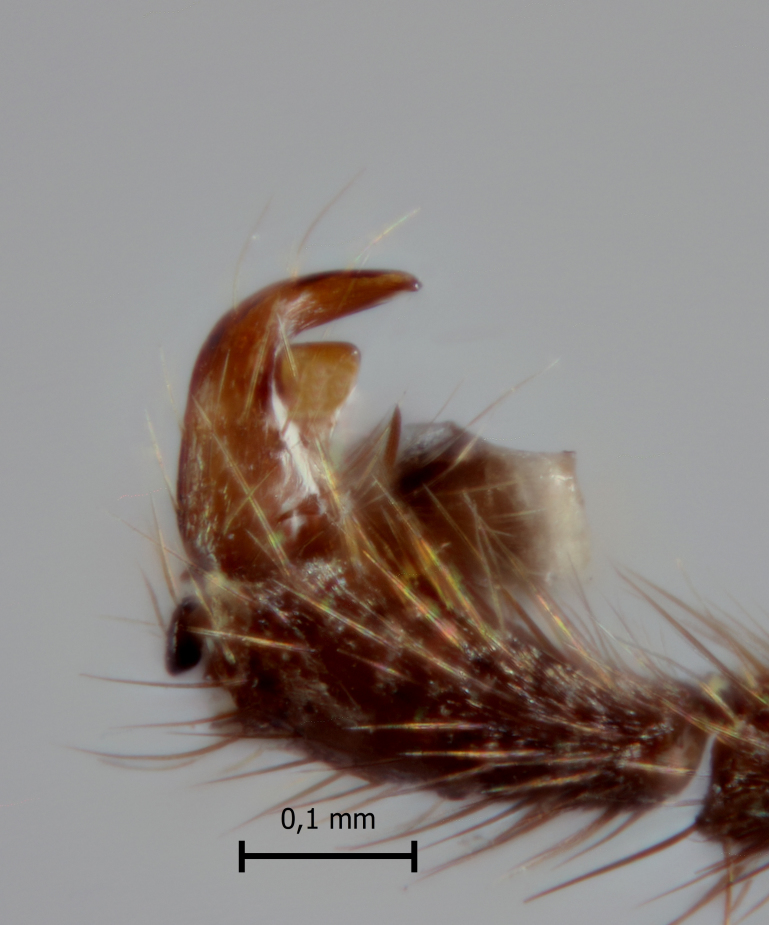
*T. soinii* Palacio et al., 2010

**Figure 4a.**
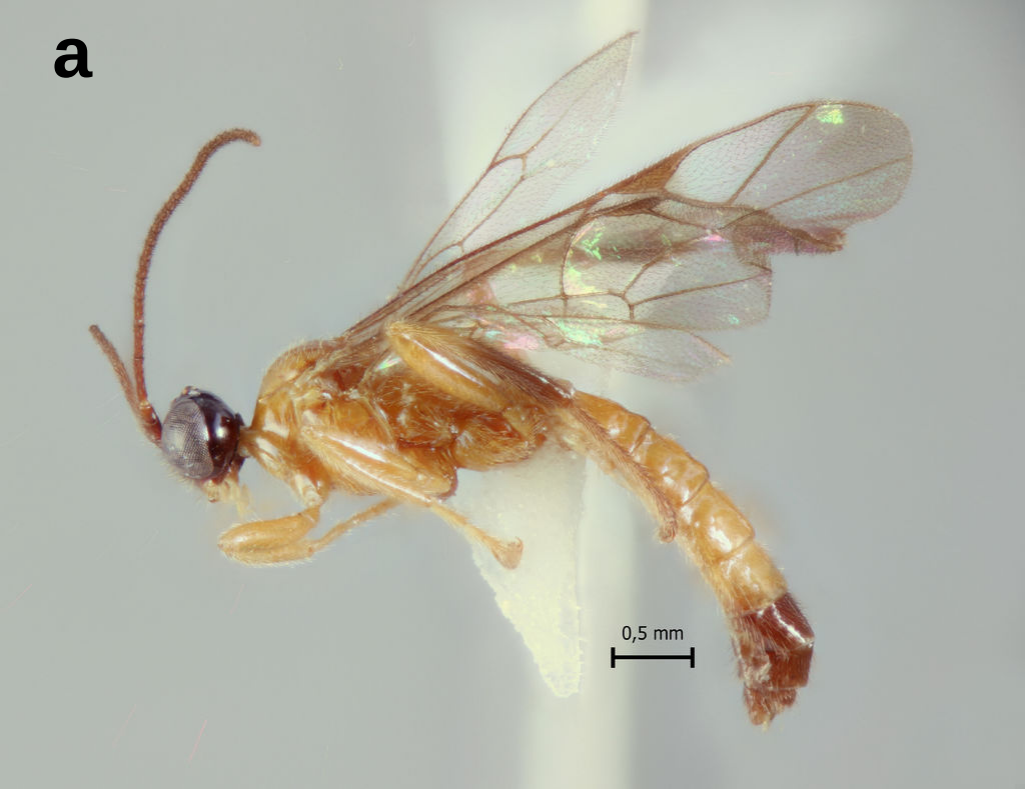
*T. amazonica* Palacio et al., 2010

**Figure 4b.**
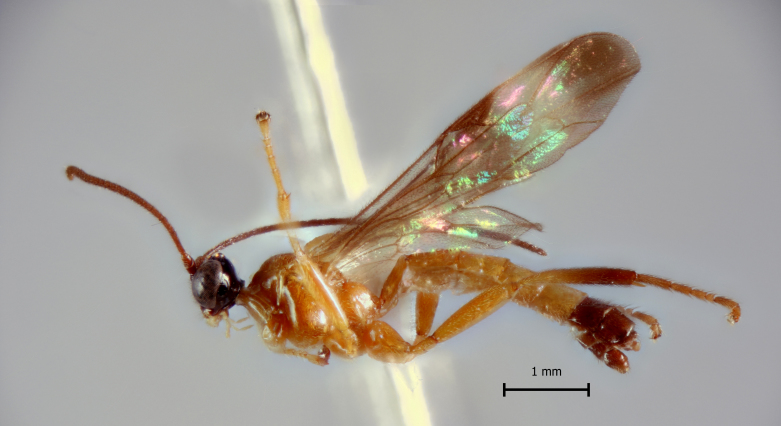
*T. carinata* Palacio et al., 2010

**Figure 4c.**
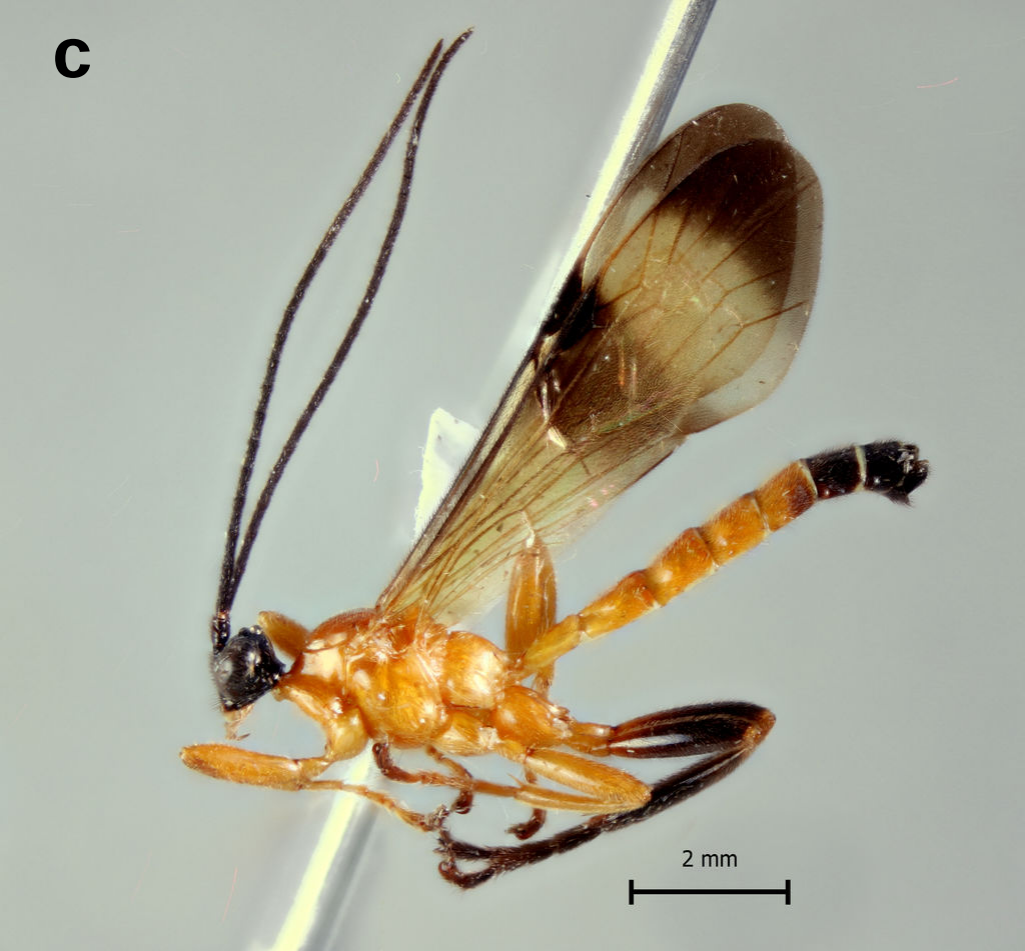
*T. matamatae* Palacio et al., 2010

**Figure 4d.**
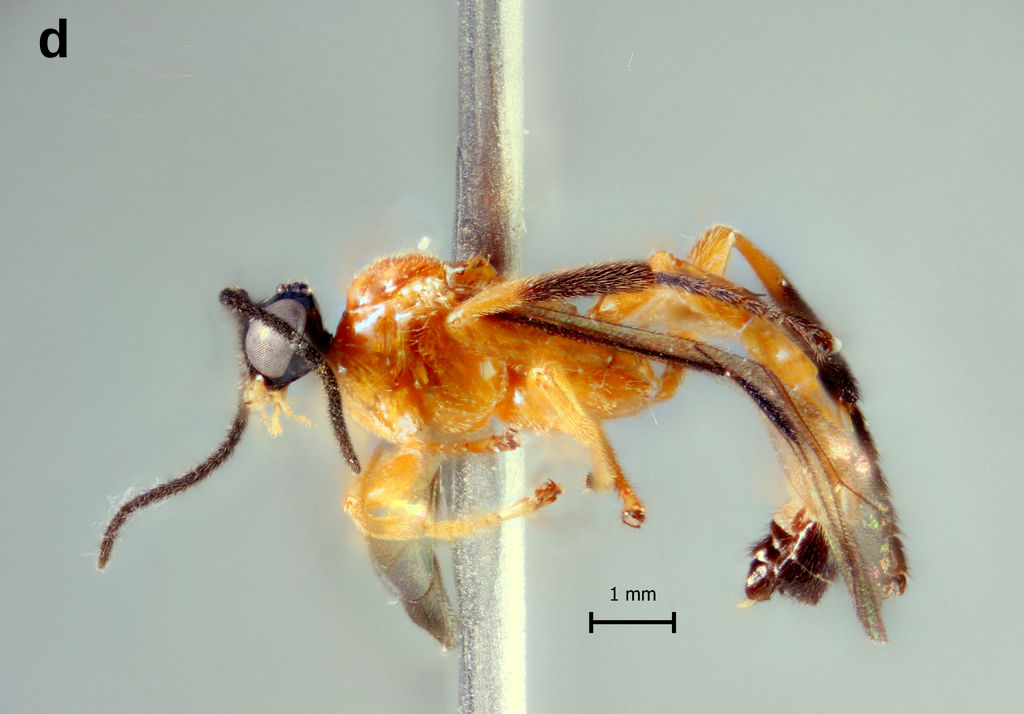
*T. soinii* Palacio et al., 2010

**Figure 5a.**
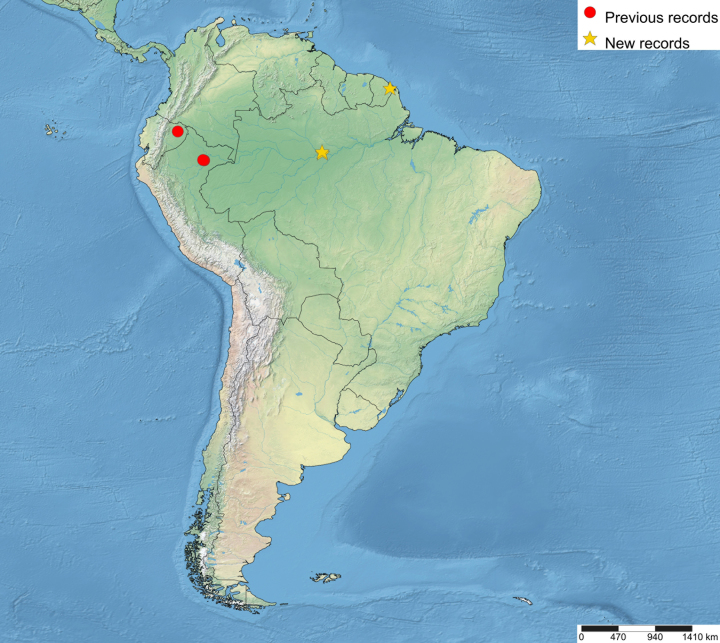
*T. amazonica* Palacio et al., 2010

**Figure 5b.**
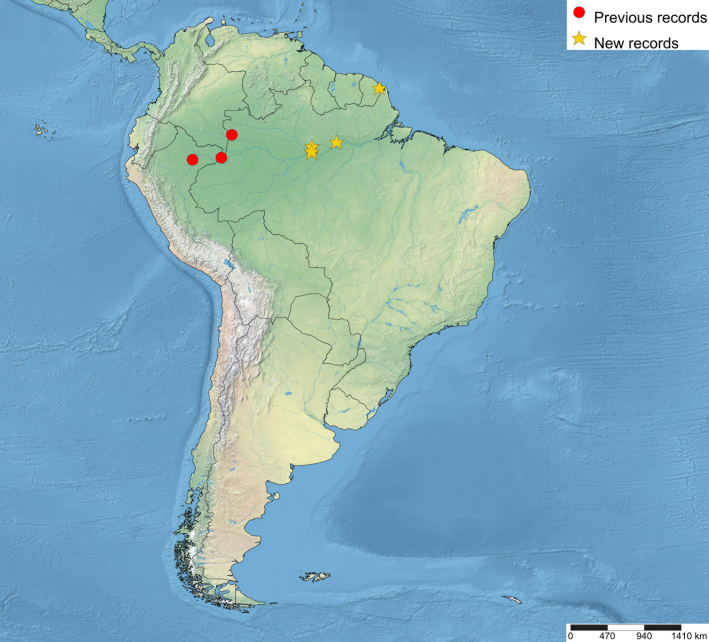
*T. carinata* Palacio et al., 2010

**Figure 5c.**
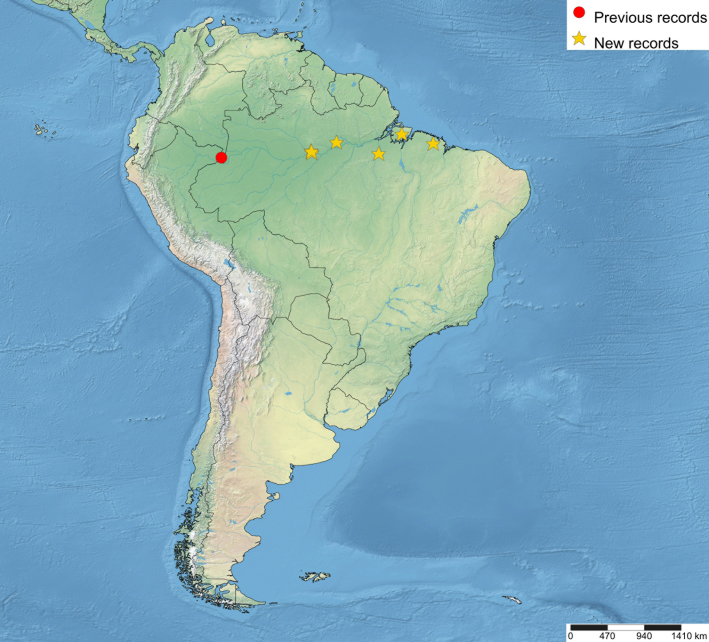
*T. matamatae* Palacio et al., 2010

**Figure 5d.**
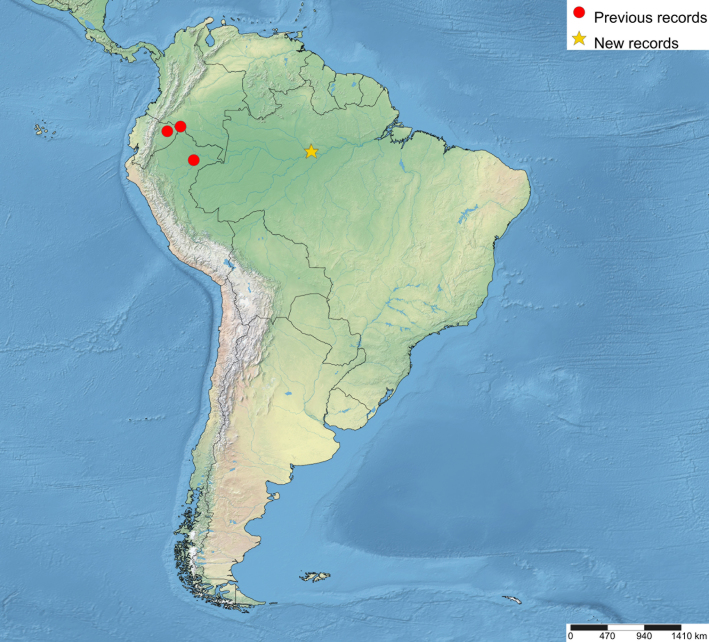
*T. soinii* Palacio et al., 2010
